# Inappropriate Use of Intravenous PPI for Stress Ulcer Prophylaxis in an Inner City Community Hospital

**DOI:** 10.4021/jocmr447w

**Published:** 2010-10-11

**Authors:** Muhammad K. Perwaiz, Gerald Posner, Fadi Hammoudeh, Frances Schmidt, Narayan Neupane, Danilo Enriquez, Neerja Gulati

**Affiliations:** a Department of Medicine, Interfaith Medical Center, Brooklyn, NY, USA; b Pulmonary division, Interfaith Medical Center, Brooklyn, NY, USA

## Abstract

**Background:**

Proton pump inhibitors (PPIs) are used for the treatment and prophylaxis of variety of acid peptic conditions including stress ulcers. There has been a persistent practice of their inappropriate use for stress ulcer prophylaxis. Purpose of our study was to measure the inappropriate use of Intravenous Proton Pump Inhibitors for stress ulcer prophylaxis and to estimate the financial burden.

**Methods:**

We carried out a retrospective, analytic study from July 2008 to June 2009 in internal medicine department. Hospital pharmacy records were used to identify all patients who received IV PPI during hospital stay. Seventy-five percent of records were randomly chosen (n=1104). PPI application was defined as indicated according to AGA guidelines.

**Results:**

Intravenous proton pump inhibitor (IV PPI) was prescribed for 68.5% of patients without any proper indication. The estimated cost of medication for inappropriate IV PPIS use during the study year was 18337 USD.

**Conclusions:**

A more rational use of PPI will have better impact on health care cost and is likely to add to patient safety.

**Keywords:**

Inappropriate use of PPI; Stress ulcer prophylaxis; Healthcare cost

## Introduction

Proton pump inhibitors (PPIs) are widely used for the treatment and prophylaxis of acid peptic conditions including stress ulcers [1].

Stress ulceration is a form of hemorrhagic gastritis that may occur following trauma or critical illness. Since the first PPI omeprazole was introduced in 1989, other drugs in the class have been marketed: esomeprazole, lansoprazole, pantoprazole and rabeprazole. There has been a substantial, continuing and unexplained rise in prescribing of PPIs. Approximately 20.5 million prescriptions were written for omeprazole in 2007. In 2007, sales of PPIs in the United States accounted for about 10 billion dollars. PPIs are therefore amongst the most abuse and misused drugs in medicine. There is growing evidence regarding safety profile and side effects of these medications [2]. Purpose of our study was to measure the inappropriate use of intravenous proton pump inhibitors (IV PPIs) for stress ulcer prophylaxis (SUP) in patients admitted to hospital (floor and ICU) and to estimate the financial burden.

## Patients and Methods

We carried out a retrospective, analytical study from July 2008 to June 2009 in our Medicine department. Written informed consent was not needed, as study was based on chart reviews only. Approval was obtained from the hospital Institutional Review Board before initiation of the study. Hospital pharmacy records were used to identify all patients who received IV PPIs during hospital stay. Esomeprazole is used as formulary PPI by the hospital.

A total of 1472 patients received IV PPI during this period, out of which 75% charts were randomly selected for review. A sequence randomizer (http://www.random.org) was used to generate a randomized sequence of patients. Patients who were already on PPI in out patient setting were excluded from the study. There was a high degree of confidence in the documentation of the indications for prophylaxis. All medical records are maintained in paper chart. All charts were thoroughly reviewed including subspecialty consults to find indication for stress ulcer prophylaxis.

The main outcome measure was the appropriateness of IV PPI use for stress ulcer prophylaxis, which was categorized as indicated, possibly indicated or not indicated: (1) Any PPI use for stress ulcer prophylaxis as per American association of gastroenterology guidelines was defined as ‘indicated’. If two indications were documented, the one that was most severe or required the longest duration of therapy was used; (2) If there was no documented indication for Stress ulcer prophylaxis, but a careful review of the chart suggested the presence of an approved indication as per AGA criteria, PPI use was categorized as ‘possibly indicated’. For example, if there was no physician-documented indication for SUP but the patient was taking an NSAID or an anticoagulant with previous history of peptic ulcer disease, PPI use was categorized as possibly indicated; (3) The remaining cases with no documented indication for use were categorized as ‘not indicated’.

Additional data abstracted from the medical charts included sociodemographic characteristics (age, sex and race), prior history of peptic ulcer disease, esophagogastroduodenoscopy or NSAIDS/steroids use, admitting diagnosis, duration of PPI use for SUP, and clinical outcome (in terms of discharge or expired). Data analysis was performed using systat version 12. Associations between appropriate PPI use for stress ulcer prophylaxis and the sociodemographic and clinical characteristics of interest were assessed using Pearson's X^2^ test.

As per guidelines from American Gastroenterology Association, following indications were considered to be acceptable for use of PPI as stress ulcer prophylaxis [3]:

1. Coagulopathy (platelet count < 50,000 mm3, INR > 1.5, or aPTT > 2 times control);
2. Mechanical ventilation for more than 48 hours;
3. History of gastrointestinal ulceration or bleeding within 1 year before admission;
4. Have at least 2 of the following risk factors: Sepsis, ICU stays longer than 1 week, occult bleeding lasting 6 days or longer, and use of more than 250 mg hydrocortisone or the equivalent.

These recommendations do not apply to patients with single-system injuries such as head trauma, spinal cord injury, or thermal injury; patients with these injuries were excluded from studies in which subjects were randomized to receive prophylaxis or no prophylaxis.

## Results

A total of 1472 patients received IV PPI during this period. And 1104 (75%) charts were randomly selected for review. Based on chart reviews, patients who received IV PPI for therapeutic indication were excluded. Approximately 713 patients (64.5%) received IV PPI for stress ulcer prophylaxis. There were no significant differences in sociodemographic characteristics between included and excluded patients (Table 1).

**Table 1:** Patients Characteristics

Characteristics	Prophylaxis (n = 713)	Treatment (n = 391)
Age, years		
Mean	59	56 (7.7)
Range	18 - 99	18 - 94
Sex, no. (%)		
Female	374 (52.4%)	191 (48.8%)
Male	339 (48.6%)	200 (51.2%)
Race, no. (%)		
Black	576 (80%)	316 (80.8%)
Hispanic	38 (5.3%)	19 (4.8%)
White	56 (7.3%)	29 (7.4%)
Asian	21 (2.9%)	11 (2.8)
Others	22 (3.0%)	16 (4.0%)
Duration of PPI use		
Mean	6.5 days	2.3 days
Range	1 - 57 days	1 - 11 days

In total, 17.3% of these patients were either admitted directly to ICU or were transferred to critical care during their hospital course (Table 2).

**Table 2:** Patients Disposition

Patient Disposition		
ICU	124	17.3%
Floor	589	82.6%

Careful data analysis showed that in 68.5% of cases IV PPIs were used without a proper indication (Table 3).

**Table 3:** Data of PPI Indication

	Indicated	Possibly-indicated	Non-indicated	
Floor	132 (22.4%)	55 (9.3%)	402 (68.4%)	589
ICU/telemetry	23 (18.5%)	10 (8.0%)	91 (73.3%)	124
Total	155 (22.1%)	65 (9.2%)	493 (68.5%)	713
Mean Age	60.6	58.4	58	
Sex				
Male	77 (48.7%)	30 (45.5%)	230 (46.6%)	
Female	81 (51.2%)	36 (54.5%)	263 (53.3%)	
Race				
Black	126 (79.7%)	54 (81.8%)	406 (82.8%)	
White	10 (6.3%)	4 (6.0%)	27 (5.3%)	
Hispanic	13 (8.2%)	7 (10.6%)	41 (8.1%)	
Asian	5 (3.1%)	1 (1.5%)	14 (2.6%)	
Medics				
NSAIDS	14 (8.8%)	10 (15.1%)	22 (4.2%)	
Steroids	7 (4.4%)	5 (7.5%)	10 (1.8%)	
Warfarin	2 (1.2%)	2 (3%)	3 (0.6%)	
Days on PPI	7.2	5.4	5	
Residents	20.9%	7.4%	69.8%	
Attending Physicians	21.8%	9.8%	67.4%	
Clinical Outcome				
Discharged	144 (91.1%)	63.3 (95.4%)	478(97.3%)	
Expired	8 (5.06%)	3 (4.5%)	13 (2.4%)	
PPI on discharge	38 (26.3%)	18 (28.5%)	78 (15.8%)	

One may infer that ICU setting and female sex itself is a risk factor for inappropriate use - ICU/telemetry: relative risk of 1.2 (95% CI of 0.84 to 1.04); Female sex: relative risk of 1.15 (95% CI 0.87 - 1.5).

There was no difference in inappropriate use between different racial groups. Prior history of NSAID use was significantly higher in possibly indicated group. There was no significant difference in inappropriate use between residents and attending Physicians.

In all these groups patients were discharged with PPI prescription, which in most cases did not have a proper indication. We were not able to discern the exact number of patients discharged on inappropriate PPI prescription as the discharge summary section did not have column for indication.

A few of the common diagnosis where IV PPIs were given for stress ulcer prophylaxis without indication are shown in Table 4.

**Table 4:** Common diagnosis for which IV PPIs was not indicated

Common Diagnosis	
Pneumonia	11.8%
Sepsis	11.7%
Acute Coronary syndrome	6.4%
UTI	6.14%
COPD Exacerbation	4.6%
Asthma exacerbation	5.1%
DVT	4.3%
Pulmonary embolism	2.3%

The cost is as following:

1. Average number of days on IV PPI = 4.5;
2. Acquisition costs of PPIs vary among institutions as a result of purchasing contracts, and cost was estimated on the basis of price provided by the pharmacy of the hospital [4];
3. Price of IV PPI (40 mg vial) = 7.5 USD;
4. Total number of non indicated PPI injections = 2445;
5. Estimated cost of medication = 18337 USD.

This estimate is only for IV PPI in an inpatient setting in an inner city community hospital. It does not include price estimation of PO PPI and cost of health care staff. If we can replicate these calculations on state or nation wide basis and do implicate the impact of documented side effects like ventilator associated pneumonia, price tag will certainly be significantly high.

## Discussion

Proton pump inhibitors are very effective agents for stress ulcer prophylaxis. Recent literature has attributed a growing number of side effects to these drugs [5]. Most widely accepted are an increased incidence of ventilator associated pneumonia, clostridium difficile infection, increased risk of fall in elderly, and many potential drug interactions[4, 6]. A more rationale and judicious use will not only prevent unnecessary health care expenditure but will definitely have a positive outcome on patient safety.

This has been a persistent practice because of physicians’ perception of the safety of this medication, lack of knowledge about practice parameters or a component of defensive medicine. Improving prescribing awareness through education and a more active involvement of clinical pharmacist could reduce inappropriate use. Our data strongly suggest the need for institutional protocols and educational interventions to promote evidence-based practice during residency training [7, 8]. Frequent review of therapy and improved communications between primary and secondary care are vital to rationalize the use of PPIs and to reduce expenditure [5].
